# Toward Precision in Biomarker Analysis: A Novel Malondialdehyde Detection Method Based on Molecularly Imprinted Polymers

**DOI:** 10.1021/acsomega.6c06926

**Published:** 2026-08-12

**Authors:** Marise Nouhra, Rita Maalouf, Simona Sawan

**Affiliations:** Department of Sciences, Faculty of Natural and Applied Sciences, 67026 Notre Dame University—Louaize, P.O. Box 72 Zouk Mosbeh, Lebanon

## Abstract

Malondialdehyde (MDA) is a critical biomarker of oxidative stress, playing a significant role in diagnosing and monitoring conditions associated with oxidative damage, including cardiovascular diseases, neurodegenerative disorders, and cancer. This study reports the synthesis and application of a molecularly imprinted polymer (MIP)-based electrochemical sensor for MDA detection using a dual biopolymer chitosan/κ-carrageenan matrix integrated with Fe_3_O_4_ nanoparticles on a boron-doped diamond (BDD) electrode. Differential pulse voltammetry (DPV) was employed to evaluate analyte recognition, while FTIR, DLS, SEM, EIS, and cyclic voltammetry were used for sensor characterization. Under optimized conditions (75% chitosan, 25% κ-carrageenan, and 10 mg Fe_3_O_4_ nanoparticles), the sensor exhibited high sensitivity (687.35 μA/μM), a low detection limit (0.005 μM), and a linear detection range of 0.026–0.26 μM. The developed platform also demonstrated good reproducibility, repeatability, and selectivity toward MDA in the presence of common interfering compounds. Analytical performance was further validated in spiked water and artificial serum samples, showing satisfactory recovery values. The proposed sensor outperformed several previously reported MDA sensing platforms, highlighting its potential as a sensitive and reliable tool for oxidative stress biomarker detection and future clinical applications.

## Graphical abstract

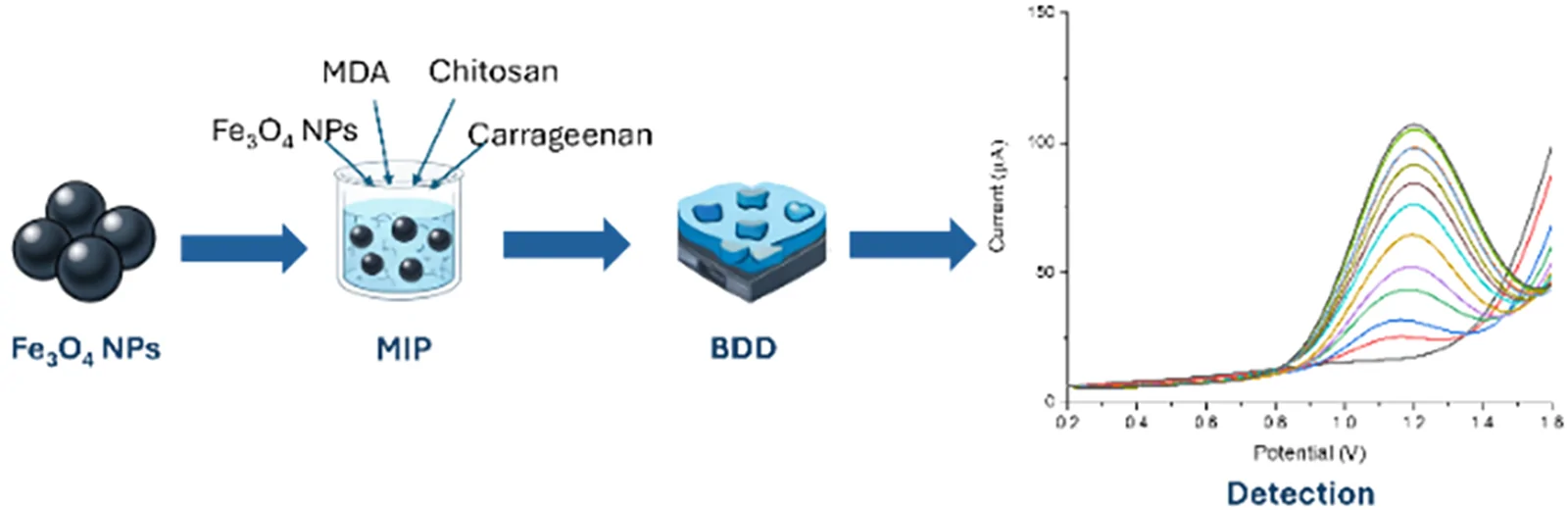

## Introduction

Oxidative stress is a key pathological mechanism underlying various chronic diseases.^1^ It is characterized by an imbalance between the production of reactive oxygen species (ROS) and the body’s antioxidant defense systems.^2^ Lipid peroxidation, a major consequence of oxidative stress, leads to the formation of MDA as a secondary metabolite.^3^ MDA’s chemical reactivity and its ability to form adducts with biomolecules such as DNA and proteins make it a critical indicator of cellular damage.^4^ Elevated levels of MDA have been implicated in the pathophysiology of several diseases, including cardiovascular diseases, diabetes, cancer, and neurodegenerative disorders.^5^ Therefore, the development of reliable and sensitive methods for detecting MDA is essential for advancing biomedical research and clinical diagnostics.

Conventional methods for MDA detection, including spectrophotometric assays and chromatography,^6^ are effective but often require extensive sample preparation, expensive equipment, and significant analysis time. Electrochemical biosensors are at the forefront of modern analytical techniques, offering significant advantages in terms of sensitivity, selectivity, and rapid detection of various analytes.^7^ These devices have gained immense popularity in diverse fields such as environmental monitoring,^8^ food safety,^9^ and medical diagnostics.^10^ Among the numerous biomolecules and markers studied using electrochemical sensors, malondialdehyde is recently gaining considerable attention.

Molecularly imprinted polymers (MIPs) have emerged as a robust strategy for enhancing the selectivity of biosensors. By mimicking the natural recognition capabilities of biological systems such as antibodies or enzymes, MIPs can create tailored binding sites for specific target molecules.^11^ This is achieved by polymerizing functional monomers around a template molecule, which is subsequently removed to leave behind a molecular imprint complementary to the target.^12^ The use of MIPs in biosensors offers several advantages, including chemical stability, low cost, and the ability to operate under harsh environmental conditions.^13^ When combined with electrochemical techniques, MIPs provide a powerful platform for developing sensors with enhanced performance characteristics.

Chitosan and carrageenan, two naturally derived biopolymers, have garnered significant attention in recent years for their roles in the fabrication of molecularly imprinted polymers, owing to their unique and complementary physicochemical properties. Chitosan, obtained through the deacetylation of chitin, a major structural component in the exoskeletons of crustaceans,^14^ exhibits exceptional biocompatibility, biodegradability, and film-forming abilities.^15,16^ Its cationic nature, attributed to the presence of primary amino groups, enables strong electrostatic interactions with negatively charged molecules,^17^ including template or functional monomers, thus facilitating the formation of stable and selective imprinting sites. Moreover, chitosan’s reactive functional groups (−NH_2_ and −OH) allow for facile chemical modifications and cross-linking,^18^ enhancing the structural tunability of the polymer network.

Carrageenan, on the other hand, is a sulfated polysaccharide extracted from various species of red algae and is widely used for its excellent gelling, emulsifying, and viscosifying properties.^19^ The presence of sulfate groups imparts an anionic character to carrageenan, making it particularly useful for modulating the electrostatic environment within the polymer matrix.^20^ Carrageenan contributes to the mechanical stability and rheological properties of composites,^21^ thus, when incorporated into MIP formulations, it aids in the formation of a uniform and robust polymer structure.

The synergistic combination of chitosan and carrageenan in MIP synthesis is particularly promising, as it leverages the electrostatic complementarity and structural compatibility of these two polymers. This blend-based-biopolymer system is expected to improve the mechanical integrity, porosity, and imprinting efficiency of the MIP, while also enhancing its affinity and selectivity toward target molecules such as malondialdehyde. Additionally, the natural origin and environmental friendliness of both chitosan and carrageenan support the development of greener and more sustainable sensing platforms.

In addition, the incorporation of nanomaterials has been shown to significantly enhance the performance of electrochemical sensors.^22,23^ Metal oxide nanoparticles, such as iron oxides, are particularly attractive due to their exceptional electrochemical properties, including high surface area, excellent conductivity, and catalytic activity.^24^ These nanoparticles facilitate efficient electron transfer processes, thereby amplifying the electrochemical signal and improving the overall performance of the biosensor. When incorporated into MIP-based biosensors, nanoparticles can significantly enhance their analytical capabilities,^25^ making them suitable for trace-level detection of analytes like MDA.

Although several electrochemical and molecularly imprinted polymer-based sensors have previously been reported for malondialdehyde detection, most systems rely on synthetic polymers, single-polymer matrices, or conventional electrode substrates. In contrast, the present study introduces a novel dual-biopolymer MIP sensing platform based on a chitosan/κ-carrageenan blend integrated with Fe_3_O_4_ nanoparticles on a boron-doped diamond (BDD) electrode.

The originality of this approach resides in the combination of three complementary components within a single sensing architecture. First, the dual-biopolymer matrix exploits the electrostatic interaction between cationic chitosan and anionic κ-carrageenan, resulting in enhanced imprinting efficiency, improved structural stability, and greater accessibility of the recognition cavities. Second, the incorporation of Fe_3_O_4_ nanoparticles promotes faster electron transfer and signal amplification, thereby improving the electrochemical response and detection sensitivity. Third, the use of a BDD electrode provides a chemically stable and highly conductive substrate with a wide potential window, further enhancing sensor performance.

The synergistic integration of these components generated a highly sensitive and selective sensing platform for MDA detection. Overall, this work presents a new MIP-based electrochemical sensing strategy that combines a tailored biopolymeric recognition matrix with a nanostructured transduction platform for enhanced malondialdehyde detection.

## Materials and Methods

### Chemical Reagents

All chemical reagents used in this study were of analytical grade and used as received without any further purification. Chitosan, κ-carrageenan, 1,1,3,3 tetramethoxypropane and all the chemicals used for interferences studies were purchased from Sigma-Aldrich. Salts used in the preparation of the phosphate buffer saline (PBS) and artificial serum as well as sulfuric acid (1%) were purchased from BDH Laboratory Supplies, while acetic acid (1%) was purchased from Scharlau. Ferric chloride hexahydrate (FeCl_3_·6H_2_O, 98%) and ferrous chloride tetrahydrate (FeCl_2_·4H_2_O, 99%) were purchased from Acros Organic. Ethanol (≥99.8%) was purchased from J. T. Baker. Distilled water with a resistivity of 18.2 MΩ·cm was used in the preparation of all solutions.

### Apparatus

FTIR spectra were obtained within the range 4000–500 cm^–1^, using a PerkinElmer Spectrum Two spectrometer. DLS measurements were performed using a Malvern ZetaSizer Nano ZSP. Samples for scanning electron microscopy were sputter-coated with a 5 nm layer of gold–palladium using a Leica EM ACE200 coater and subsequently examined with a Hitachi TM1000 SEM at an acceleration voltage of 4 kV.

All electrochemical measurements were conducted using a PalmSens4 with PS trace software. A conventional three electrode system was employed comprising a modified boron-doped diamond as the working electrode (0.07 cm^2^ of active surface, determined by an O-ring seal), platinum plate as a counter electrode (0.19 cm^2^ of active surface, determined by an O-ring seal), and Ag/AgCl as a reference electrode. All electrochemical measurements were conducted in 0.1 M PBS pH 7.4.

### Synthesis of Fe_3_O_4_ Nanoparticles

Magnetic nanoparticles were synthesized as previously reported by Sawan et al.^26^ utilizing a modified Massart’s method.^27^ This method involves the coprecipitation of an aqueous mixture of ferric and ferrous salt hydrates followed by the addition of a base. Briefly, 2.7 g of FeCl_3_·6H_2_O and 1.2 g of FeCl_2_·4H_2_O were mixed with a molar ratio of 2:1 and dissolved in 90 mL of deionized water. Afterward, a 100 mL volume of a 1 M NaOH solution was slowly added dropwise, with vigorous stirring and under a nitrogen atmosphere at 0 °C. The change in color indicated the formation of iron oxide nanoparticles. The resulting solution underwent magnetic stirring for 4 h to ensure the nucleation and growth of magnetite nanoparticles. These nanoparticles were subsequently collected using an external magnet, after which they underwent multiple washing steps with water and ethanol and were ultimately dried under vacuum.

### Fabrication of the Electrochemical MIP/Fe_3_O_4_ NPs/BDD Electrode

Cyclic voltammetry (CV) measurements were carried out to investigate the electrochemical behavior of MDA. Electrochemical Impedance spectroscopy (EIS) measurements were carried out under an open-circuit voltage, with the frequency range of 0.1 Hz–1 MHz. Differential pulse voltammetry (DPV) was employed to evaluate MDA binding and elution, as well as the sensitivity, reproducibility, stability, and reusability of the sensor. DPV measurements were conducted over a potential range of 0–2 V using a modulation amplitude of 50 mV, a pulse width of 100 ms, and a step potential of 5 mV. These parameters were selected following preliminary optimization studies in which modulation amplitude, pulse width, and step potential were systematically varied to optimize peak definition, signal intensity, and signal-to-noise ratio. All electrochemical measurements were performed at room temperature.

Prior to modification, the BDD electrodes were cleaned by ultrasonication in acetone for 15 min, followed by rinsing with Milli-Q water. The electrodes were then immersed in freshly prepared aqua regia (HCl/HNO_3_ 3:1 v/v) for 3 min to remove organic residues and adsorbed contaminants. After acid treatment, the electrodes were thoroughly washed with Milli-Q water and ethanol, then dried under a nitrogen stream. A chitosan/MDA solution (2:1 ratio) containing Fe_3_O_4_ nanoparticles was subsequently mixed with a carrageenan solution directly on the BDD electrode surface. The resulting mixture was allowed to polymerize during drying, leading to the formation of the molecularly imprinted polymer (MIP) layer. The template molecules were then removed by washing the electrode with 1% acetic acid for 20 min under constant stirring. Figure 1 presents a schematic illustration of the synthesis procedure for the MIP/Fe_3_O_4_ NPs/BDD electrode. The nonimprinted polymer (NIP/Fe_3_O_4_ NPs/BDD) electrode was prepared under identical conditions, except that MDA molecules were omitted, to enable comparative analysis.

**Figure 1:**
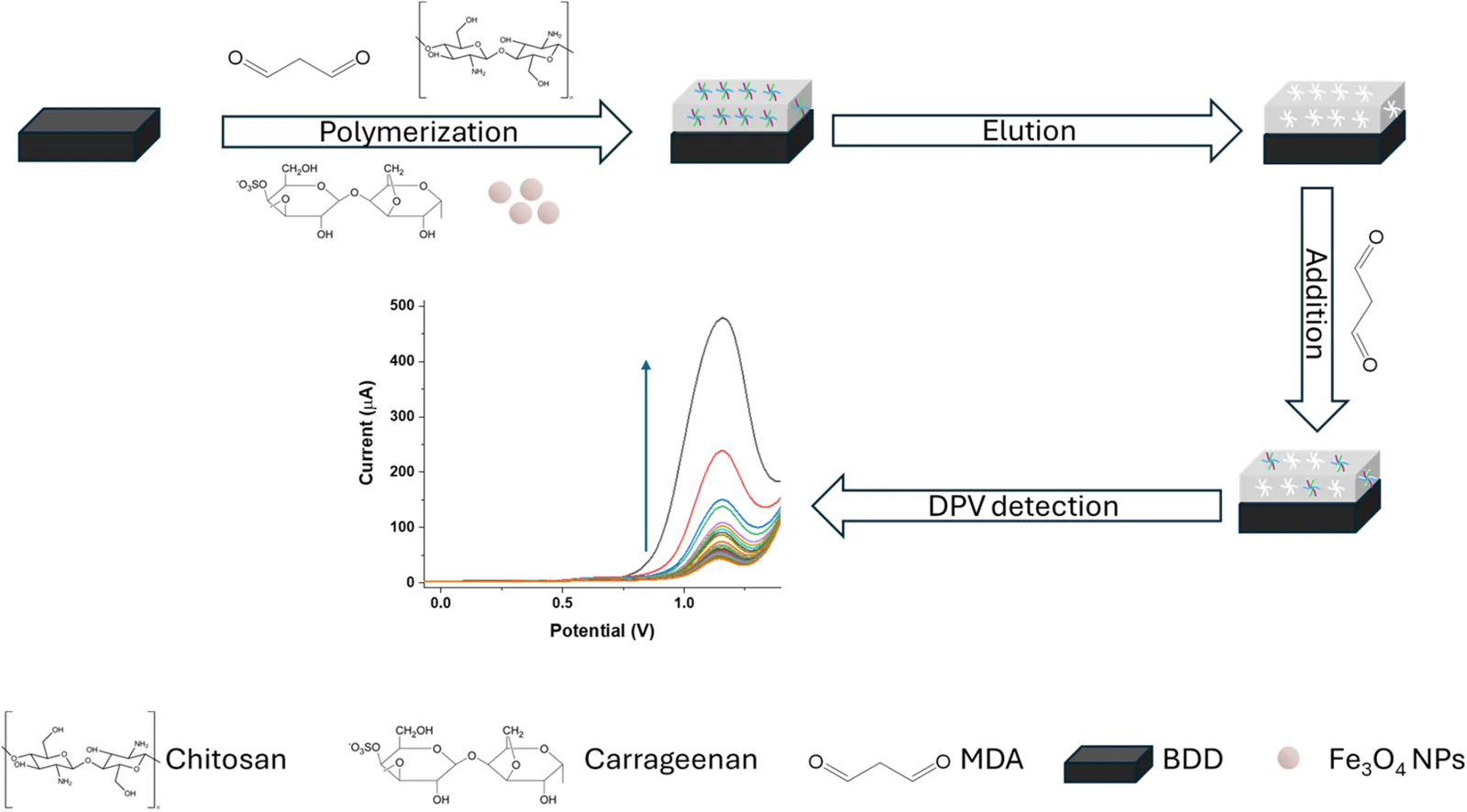
Scheme representing the synthesis of MIP/Fe_3_O_4_ NPs/BDD.

### Electrochemical Measurements

#### Reproducibility, Repeatability, and Selectivity of the MIP/Fe_3_O_4_ NPs/BDD Sensor

The reproducibility of the sensor was evaluated using three independently prepared MIP/Fe_3_O_4_ NPs/BDD electrodes in the presence of 0.05 μM MDA, while repeatability was assessed through 11 consecutive DPV measurements using the same electrode, with regeneration using 1% acetic acid after each cycle. To evaluate the selectivity of the proposed sensor toward MDA, the binding response of structurally related compounds and commonly occurring ions in biological samples was investigated using the MIP/Fe_3_O_4_ NP polymer. Glucose, acetone, urea, ascorbic acid, Na^+^, K^+^, and Ca^2+^ were selected as potential interfering species and tested under optimized conditions at a concentration of 1 mM in the presence of 0.05 μM MDA.

#### Analytical Application of the MIP/Fe_3_O_4_ NPs/BDD Electrode

To confirm its practical applicability, the optimized MIP/Fe_3_O_4_ NPs/BDD sensor was employed for the detection of MDA in spiked water samples and artificial serum. Briefly, each sample was spiked with MDA at concentrations of 0.05 and 0.1 μM, followed by differential pulse voltammetry (DPV) measurements for MDA quantification.

## Results and Discussion

The synthesized materials were characterized using FTIR to confirm the successful formation of the MIP and NIP, SEM to assess the morphology of the polymers, and DLS to evaluate the surface charge and size of the imprinted materials.

### FTIR Characterization of MIP and NIP

Figure 2 presents the infrared spectra of the MIP (spectrum A) and NIP (spectrum B). The spectra exhibit high similarities with slight differences pertaining to the intensities of specific peaks. The successful incorporation of iron oxide nanoparticles in both is indicated by a peak at 580 cm^–1^ corresponding to the vibration of Fe–O bond.^28^

**Figure 2:**
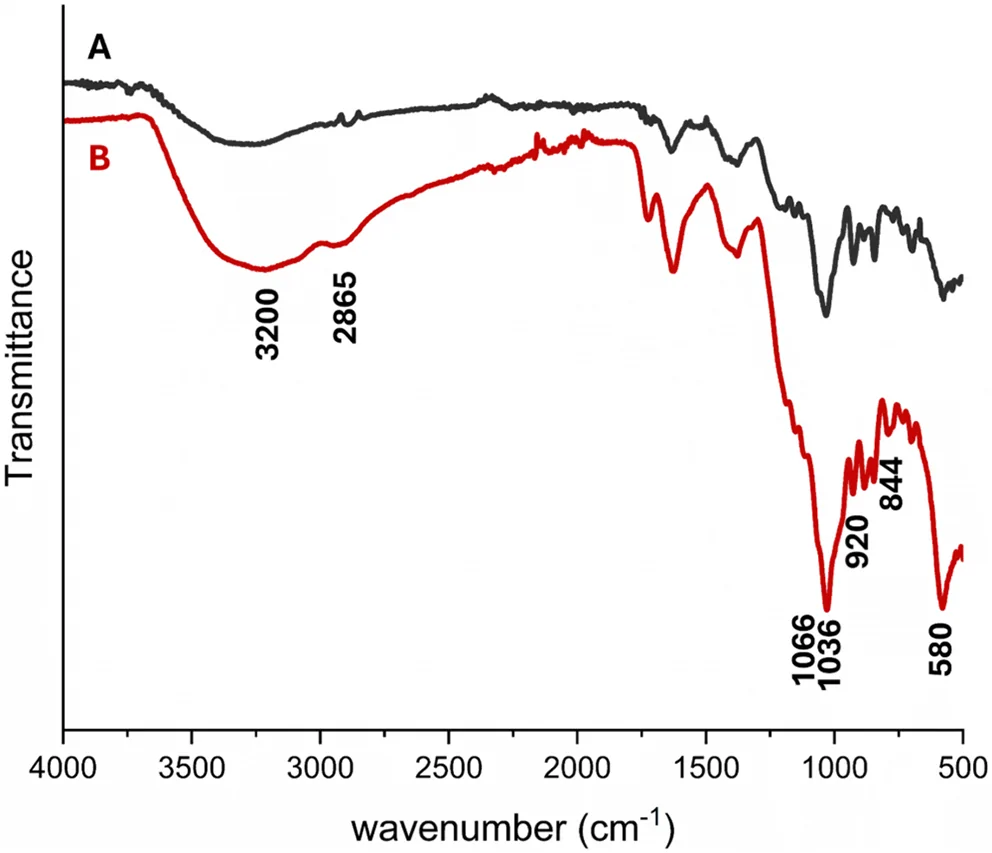
FTIR spectra of (A) MIP/Fe_3_O_4_ and (B) NIP/Fe_3_O_4_.

A peak at 920 cm^–1^ characteristic of C–O–C 3,6-anhydro-o-galactose of carrageenan, along with the peaks at 844 cm^–1^ corresponding to C–O–SO_3_ stretching and at 1066 and 1036 cm^–1^ corresponding to C–O–H^29^ prove the successful polymerization of the 2 polysaccharides. Stabilization of the proposed polymers is most likely due to electrostatic interactions between the amide/amino groups of chitosan and the sulfate groups of carrageenan.^30^ The formation of the polymeric network results primarily from polyelectrolyte complexation between protonated amino groups of chitosan and negatively charged sulfate groups of κ-carrageenan. Additional hydrogen bonding involving hydroxyl and amino functionalities contributes to the stabilization of the three-dimensional matrix.

The NIP shows a pronounced peak at 2865 cm^–1^, corresponding to C–H stretching in CH_2_, and a broad peak around 3200 cm^–1^, associated with O–H and N–H stretching. These peaks are less intense in the MIP, indicating that MDA was successfully imprinted, as its interaction with the polymer reduces the intensity of these vibrations.

Accordingly, these results indicate that the molecular recognition between malondialdehyde and the chitosan/κ-carrageenan-based MIP is governed primarily by noncovalent interactions. During the imprinting process, hydrogen bonding plays a dominant role, occurring between the carbonyl groups (−CHO) of MDA and the amino (−NH_2_) and hydroxyl (−OH) functional groups of chitosan, as well as the hydroxyl groups of κ-carrageenan. In addition, electrostatic interactions contribute to the stabilization of the polymer matrix, where protonated amino groups of chitosan (−NH_3_^+^) interact with the negatively charged sulfate groups (−SO_3_^–^) of carrageenan, creating a structurally stable and electrostatically favorable environment around the template molecule.

These combined interactions enable the formation of highly specific binding cavities that are complementary in size, shape, and functional group arrangement to MDA. After template removal, these cavities retain their recognition ability, allowing selective rebinding of MDA through the same hydrogen bonding and electrostatic interactions. The imprinting process is therefore based on a noncovalent mechanism, which facilitates reversible binding and efficient sensor regeneration.

### DLS Characterization

DLS was used to measure the hydrodynamic diameter of the NIP and MIP samples using deionized water as a dispersant. The measured average particle size (*Z*-average) for the MIP was approximately 457 nm, whereas the NIP exhibited a smaller size of around 274.1 nm. The smaller NIP size is likely due to varying interactions between template and biopolymers.^31^ Those interactions affect particle nucleation and growth leading to a larger MIP size compared to the NIP.^32^ Both samples exhibited low polydispersity index (PDI) values less than 0.2, indicating a uniform particle size distribution and minimal aggregation, which are desirable characteristics for consistent performance in sensing. The larger size of the MIP suggests successful template incorporation and formation of imprinted cavities, which may contribute to enhanced binding capacity and improved sensor sensitivity.

### SEM Characterization

Figure 3 presents SEM images illustrating the surface morphology of the synthesized MIP and NIP materials. At a magnification of 1000×, it is possible to observe that the formed polymers have relatively uniform morphologies and a porous texture. Moreover, the similar morphology of MIP and NIP indicates that imprinting primarily affects chemical recognition rather than bulk structure, confirming that selective binding arises from functional cavity formation rather than morphological changes.^33^

**Figure 3:**
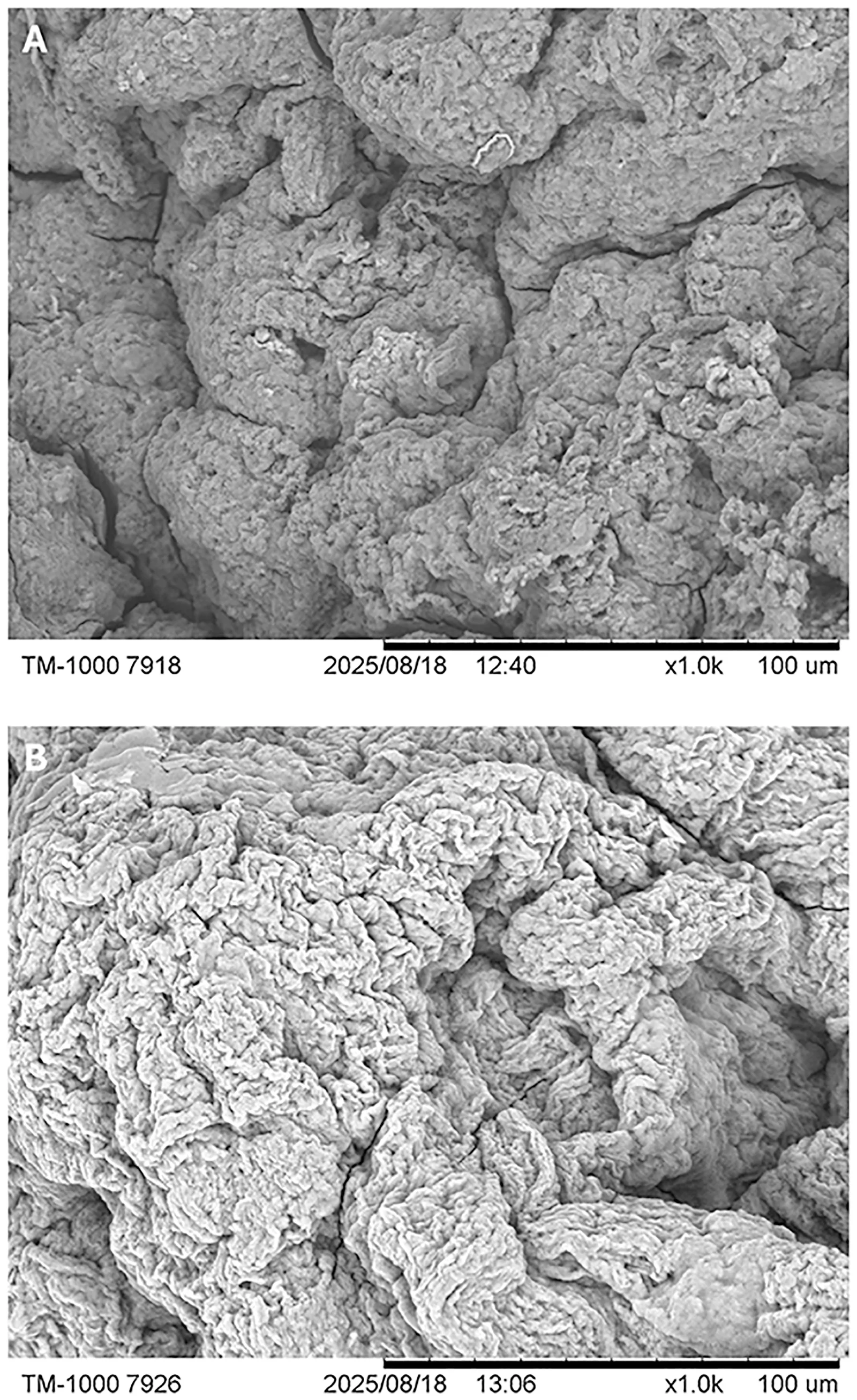
SEM images of (A) MIP and (B) NIP.

### Electrochemical Analysis

#### Electrochemical Properties of MDA

To investigate the electrochemical behavior of malondialdehyde, cyclic voltammetry measurements were recorded in the range from 0 to 2 V at a scan rate of 80 mV/s. A scan rate of 80 mV s^–1^ was selected based on preliminary optimization experiments, as it provided a well-defined oxidation peak with good peak resolution while minimizing capacitive current contributions. Figure 4 shows cyclic voltammograms at bare BDD electrode in 0.1 M phosphate buffer solution pH = 7.4 in the absence and presence of MDA. Whereas no peaks appear in the absence of MDA, a redox behavior was observed at 1.2 V after the addition of 1 mM of MDA. The observed peak corresponds to the irreversible oxidation of MDA on a bare BDD electrode vs Ag/AgCl electrode.

**Figure 4:**
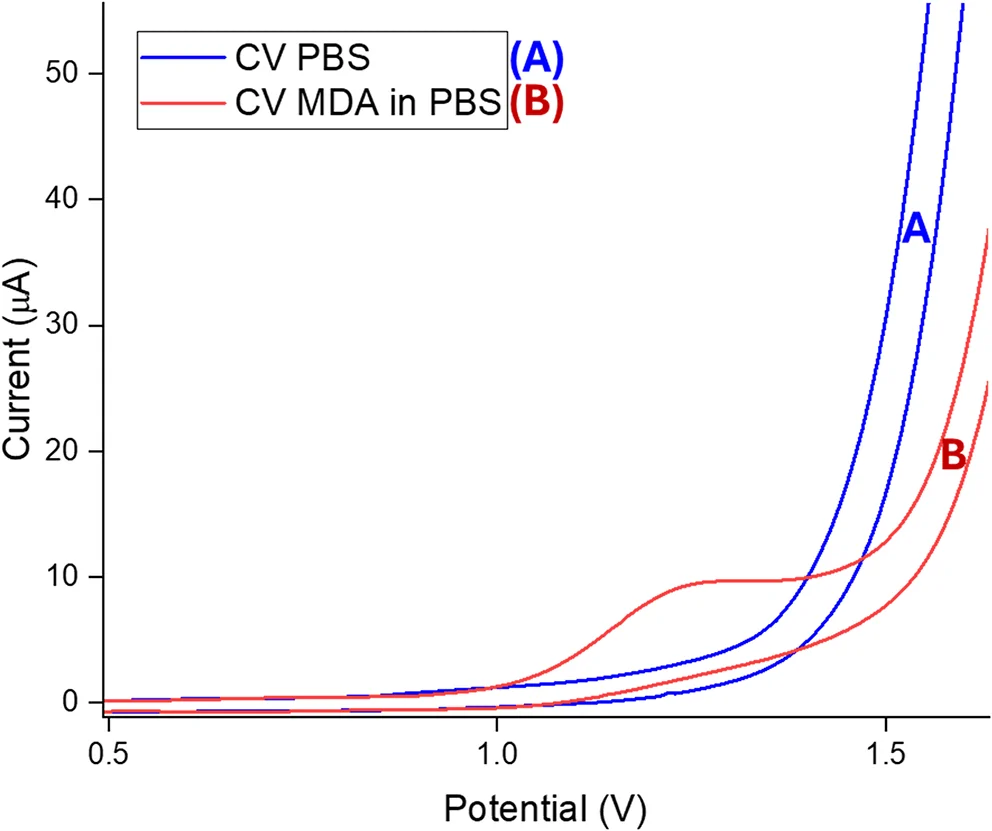
Cyclic voltammograms in the absence (A) and presence (B) of 1 mM MDA in 0.1 M PBS pH 7.4.

#### Electrochemical Properties of Electrodes

The electrochemical properties of the electrodes were evaluated by electrochemical impedance spectroscopy (EIS) in a 5 mM [Fe­(CN)_6_]^3–^/^4–^ solution containing 0.1 M KCl, as shown in Figure 5. The charge transfer resistance (*R*_ct_) decreased progressively from 451 Ω for the bare BDD electrode to 329 Ω after modification with the chitosan/κ-carrageenan film and further to 149 Ω following incorporation of Fe_3_O_4_ nanoparticles. This marked reduction in *R*_ct_ confirms that the successive surface modifications substantially facilitated interfacial electron transfer. The improved charge transfer kinetics can be attributed to the enhanced electrical conductivity and increased electroactive surface area provided by the composite coating, which lowers the energy barrier for electron transfer and promotes more efficient electrochemical reactions.

**Figure 5:**
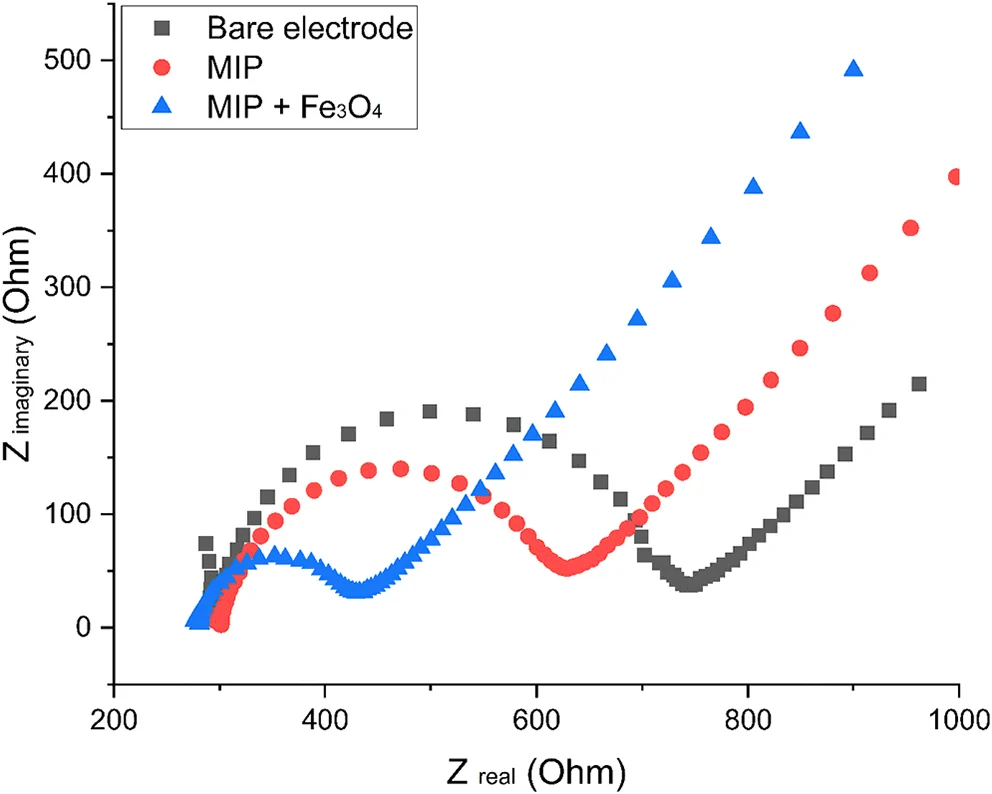
Electrochemical impedance spectroscopy Nyquist plots of the bare BDD electrode and the BDD electrodes modified with chitosan/κ-carrageenan and chitosan/κ-carrageenan/Fe_3_O_4_.

#### Regeneration of the MIP/Fe_3_O_4_ NPs/BDD Electrode

The efficiency of the elution process was examined by monitoring the differential pulse voltammetry peak current response of the MIP/Fe_3_O_4_ nanoparticles before and after the removal of malondialdehyde. As illustrated in Figure 6, a distinct oxidation peak for MDA was observed at 0.94 V in PBS at pH 7.4, confirming its successful binding to the MIP layer. Upon treatment with 1% acetic acid for 20 min, this oxidation peak completely disappeared, indicating that MDA was effectively eluted from the recognition sites. This not only confirms the efficient removal of the analyte but also demonstrates the successful regeneration of the MIP/Fe_3_O_4_-based sensor for potential reuse.

**Figure 6:**
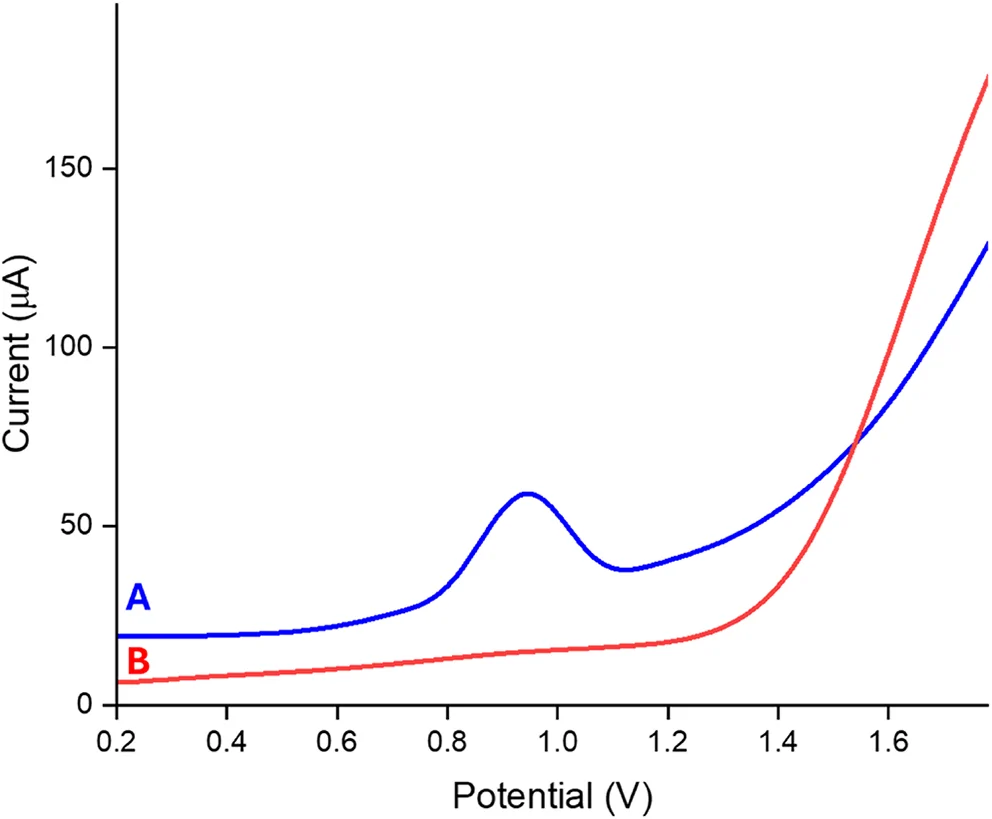
Differential pulse voltammetry (A) MIP with MDA and (B) MIP after elution of MDA using 1% acetic acid.

#### Experimental Parameters Optimization

To design a better electrochemical sensor based on molecularly imprinted polymers, some parameters need to be evaluated. These parameters include the ratio between the biopolymers, the amount of materials deposited on the electrode, and the amount of nanoparticles added.

#### Optimal Ratio between the Chitosan and Carrageenan

The quantity of imprinted cavities and the thickness of the resulting polymer film formed during polymerization are influenced by the molar ratio between the template molecule and the biopolymers.^34,35^ This ratio also has a significant impact on the redox peak current, reflecting changes in the electrochemical properties of the imprinted polymer. Since 2 polysaccharides were chosen as biopolymers for this MIP, the ratio between them was optimized first in order to develop a sensor with the greatest affinity toward MDA.

In order to determine the optimal amount of chitosan and κ-carrageenan that should be used for the formation of the MIP, the following ratios of chitosan: κ-carrageenan were tested: 1:0, 3:1, 1:1, 1:3 and 0:1. The maximum peak current response was obtained for the 3:1 ratio had the best sensitivity and limit of detection compared to other ratios (Figure 7). Those findings are in accordance with previous reports that proved that a higher percentage of chitosan compared to carrageenan improves the electrochemical signal because of the charge interactions between the polysaccharides and the analyte.^30^ The synergistic integration of the cationic chitosan and anionic κ-carrageenan created a structurally stable and electrostatically favorable polymer network, enabling the formation of well-defined recognition cavities specific to MDA. The incorporation of Fe_3_O_4_ nanoparticles significantly improved electron transfer efficiency and signal amplification, resulting in enhanced analytical performance. Fe_3_O_4_ nanoparticles interact with hydroxyl, amino, and sulfate functionalities of the biopolymers through hydrogen bonding and surface coordination interactions. These interactions improve nanoparticle dispersion, increase effective surface area, facilitate electron transfer, and enhance catalytic activity toward MDA oxidation.

**Figure 7:**
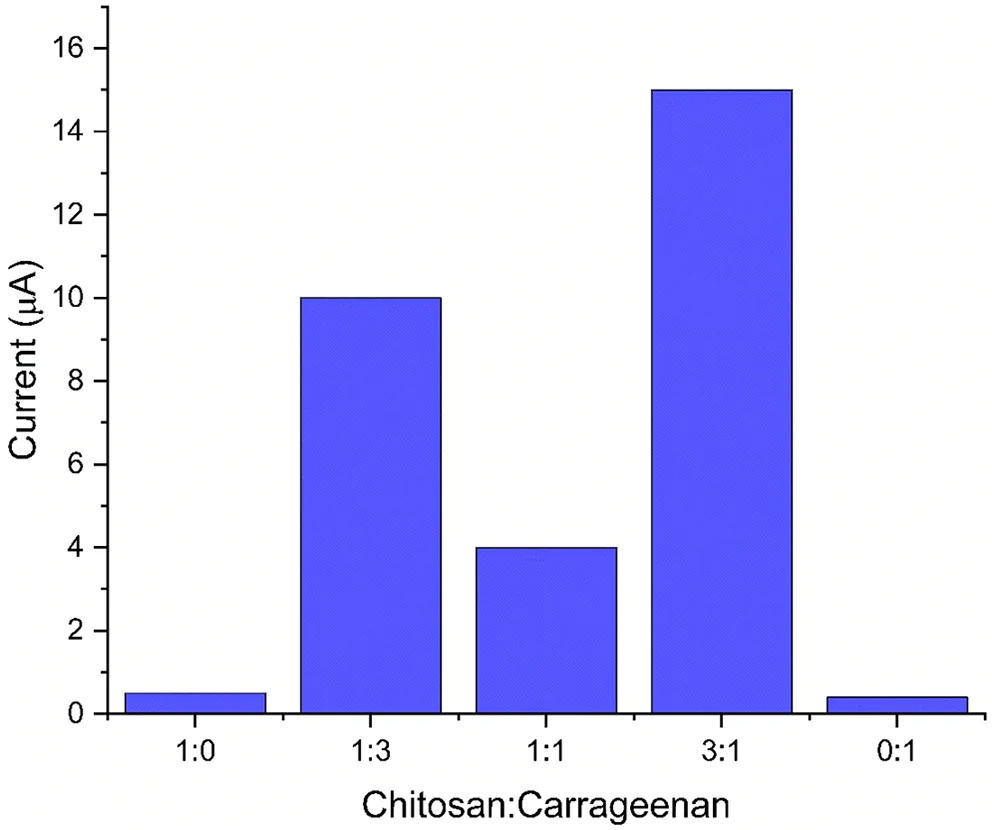
Average peak current response as a function of the different chitosan/carrageenan ratios.

#### Optimal Deposition Volume on the Electrode

The volume of chitosan/κ-carrageenan that should be applied on the BDD electrode was also investigated. The volumes tested varied between 5, 10, and 20 μL. The oxidation peak of MDA using 10 μL of the MIP on the electrode had the greatest intensity. This can be explained by the balance between electrode coverage and MIP thickness. When only 5 μL was applied, the MIP coverage was likely insufficient, offering fewer available recognition sites and thus limited interaction with MDA. On the other hand, 20 μL lead to the formation of a thicker polymeric layer that possibly hindered electron transfer and slowed the diffusion of the analyte to the electrodes surface. At 10 μL, the electrode surface was optimally coated, providing a sufficient number of imprinted sites while maintaining efficient charge transfer and analyte accessibility.^36,37^

Accordingly, 10 μL was selected as the optimal deposition volume. This volume was composed of 7.5 μL of the chitosan/MDA solution (prepared in a 2:1 ratio) and 2.5 μL of κ-carrageenan, which were deposited onto the BDD electrode to form the molecularly imprinted polymer layer for subsequent electrochemical measurements.

#### Evaluation of the MIP/Fe_3_O_4_/BDD Electrode Analytical Performance

To investigate the analytical performance of the MIP/Fe_3_O_4_ NPs/BDD electrode, DPV measurements conducted in the presence of increasing concentrations of MDA yielded a well-defined anodic peak at a potential of 1.1 V vs Ag/AgCl. A constant stirring interval was maintained between each MDA addition. As shown in Figure 8a, oxidation current increases linearly with increasing concentrations of MDA in the range of 0.026 μM to 0.26 μM. Different masses of NPs (0, 5, 10, and 15 mg) were assessed, leading to various calibration curves (Figure 8b–e).

**Figure 8:**
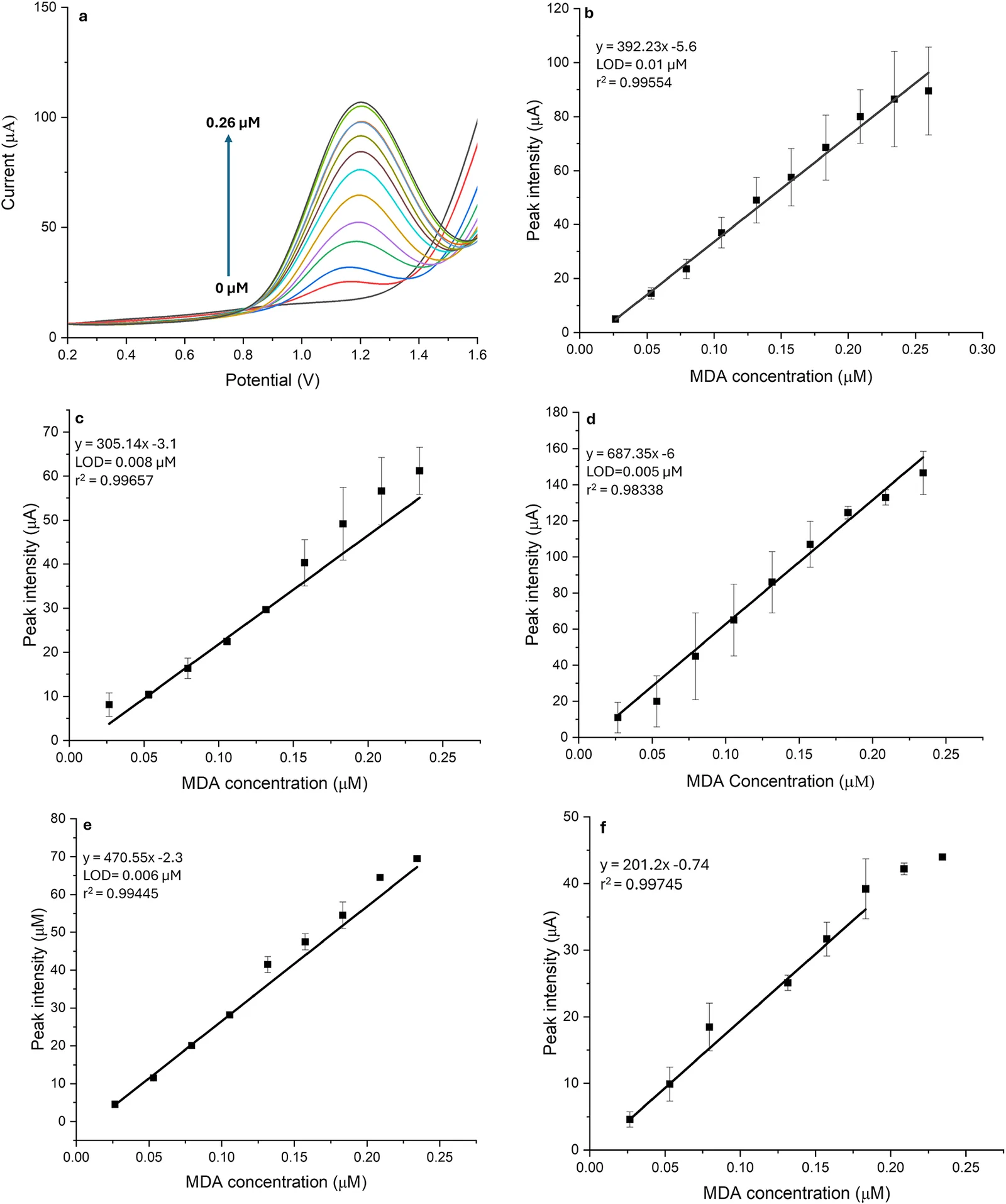
(a) DPV of MDA at the MIP/Fe_3_O_4_ NPs/BDD electrode over a concentration range of 0–0.26 μM, linear calibration ranges at the (b) MIP/BDD, (c) MIP/5 mg Fe_3_O_4_ NPs/BDD, (d) MIP/10 mg Fe_3_O_4_ NPs/BDD, (e) MIP/15 mg Fe_3_O_4_ NPs/BDD, and (f) NIP/Fe_3_O_4_ NPs/BDD electrodes.

The best analytical performance was observed when 10 mg of Fe_3_O_4_ NPs were used (Figure 7d). The corresponding linear equation is *I* (μA) = 687.35 *C* (μM) – 5.97 with a coefficient of determination *r* ^2^ of 0.98338. The MIP-based sensor exhibited a sensitivity of 687.35 μA/μM, indicating its capacity to reliably respond to changes in MDA concentration. Additionally, the calculated limit of detection (LOD) was found to be 0.005 μM using the standard deviation of the blank, such that the LOD = 3.3 × standard deviation/slope of the calibration curve, underscoring the sensor’s ability to detect MDA even at low concentrations. In comparison to the optimum MIP/Fe_3_O_4_ NPs/BDD sensor, the NIP/Fe_3_O_4_ NPs/BDD sensor showed a narrower linear range (0.026 to 0.18 μM), and a lower sensitivity (201.2 μA/μM) as depicted in Figure 7f. This difference highlights the crucial role of molecular imprinting in enhancing sensor performance. The absence of specific recognition cavities in the NIP restricts selective binding of MDA, resulting in weaker electrochemical responses and reduced analytical efficiency. The calculated imprinting factor of 3.18 further confirms the contribution of the imprinting process, demonstrating that the MIP electrode response is more than three times higher than that of its nonimprinted counterpart. These results validate the successful fabrication of a selective sensing platform and emphasize the advantage of combining molecular imprinting with Fe_3_O_4_ nanoparticle incorporation for sensitive MDA detection.

Examining extensive research related to MDA sensors, the performance of the developed sensor was compared with previous studies as shown in Table 1. This table presents the performance of recent electrochemical sensors for the detection of MDA. The proposed sensor exhibits significantly higher sensitivity than previously reported systems, likely due to the synergistic effect of the dual-biopolymer matrix and Fe_3_O_4_ nanoparticles, which enhance both binding efficiency and electron transfer. The relatively narrow linear range compared with some previously reported sensors is likely attributable to the finite number of accessible imprinted recognition sites within the polymer matrix. As the analyte concentration increases, progressive occupation of these binding sites may lead to partial saturation, resulting in deviations from linearity at higher concentrations.

**Table 1:** Comparison with Various Electrochemical Sensing Platforms for the Detection of Malondialdehyde^a^

Electrode	Method	Linear range (μM)	LOD (μM)	Sensitivity (μA/μM)	Reference
Poly(DA-CS)-AgNPs-GCE	SWV	1.45–7.9	0.047	0.9634	^38^
RF-PT-AgNPs/GCE	DPV	89–1680	–	–	^39^
TCPP–MGO/SPCE	DPV	0.01–100	0.007	0.1604	^40^
MGO@MIPy/SPCE	DPV	0.01–100	0.003	–	^41^
PT/Au electrode	DPV	0. 005–3	–	0.172	^42^
MIP/Fe_3_O_4_ NPs/BDD	DPV	0.026–0.26	0.005	687.35	This work

a Poy­(DA-CS): polydopamine-chitosan, RF-PT: self-assembled riboflavin-taurine, AgNPs: silver nanoparticles, GCE: Glassy carbon electrode, TCPP-MGO: porphyrin-functionalized magnetic graphene oxide, SPCE: screen printed carbon electrode, MIPy: molecularly imprinted polypyrrole, PT: polytaurine, Au: gold, SWV: square wave voltammetry Evaluation of Sensor Reproducibility, Repeatability, and Selectivity.

Although some reported sensors exhibit wider linear ranges or lower limits of detection, the proposed sensor offers several advantages, including the use of sustainable biopolymer-based recognition elements, a straightforward fabrication process, high analytical sensitivity, excellent regeneration capability, and the exceptional chemical and electrochemical stability of the BDD electrode.

#### Evaluation of Sensor Reproducibility, Repeatability, and Selectivity

The reproducibility of the developed MIP/Fe_3_O_4_ NPs/BDD sensor was evaluated using differential pulse voltammetry (DPV) measurements obtained from three independently fabricated electrodes prepared under identical conditions in the presence of 0.05 μM MDA. The fabricated sensors exhibited excellent reproducibility, with a relative standard deviation (RSD) of 4.6%. This low variability highlights the reliability of the fabrication protocol and suggests a homogeneous distribution of Fe_3_O_4_ nanoparticles and imprinted recognition sites within the polymer matrix. These findings confirm the robustness and consistency of the developed sensing platform for electrochemical detection of MDA.

The repeatability of the sensor was further assessed by performing 11 consecutive DPV measurements in 0.1 M PBS (pH 7.4) containing 0.05 μM MDA. Following each measurement, the sensor surface was regenerated using 1% acetic acid. The results obtained demonstrated good operational stability, with an RSD value of 7.5% over the 11 cycles. The sensor maintained stable electrochemical performance throughout repeated use, with recovery values ranging from 100% to 121%. These findings indicate that the molecularly imprinted polymer retained the integrity of its recognition cavities after repeated regeneration cycles, supporting its suitability for long-term and practical analytical applications.

The selectivity of the proposed sensor was investigated by recording the DPV responses in the presence of several potential interfering substances commonly found in biological samples, including acetone, urea, ascorbic acid, glucose, Na^+^, K^+^, and Ca^2+^, each at a concentration of 1 mM. The responses were compared with that obtained for 0.05 μM MDA using the MIP/Fe_3_O_4_ NPs/BDD electrode. The results demonstrated that these interfering compounds produced minimal effects on the MDA signal, despite being present at concentrations substantially higher than that of the target analyte. Variations in the recorded current responses remained within ± 10% relative to the MDA signal, as illustrated in Figure 9. These findings confirm the high selectivity of the developed sensor toward MDA and demonstrate its capability for reliable detection in complex biological matrices.

**Figure 9:**
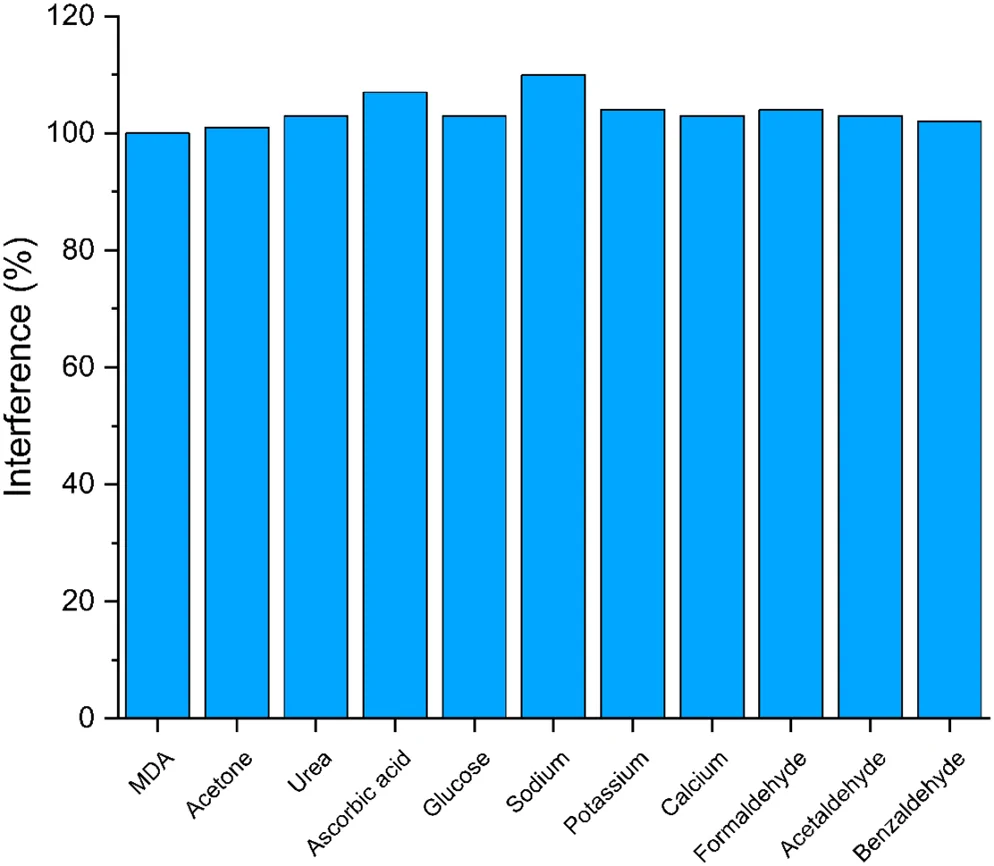
Current response signals obtained by different interfering molecules and ions.

#### Application of the MIP/Fe_3_O_4_ NPs/BDD Sensor in Spiked Real Samples

To evaluate the practical applicability of the proposed sensor, its analytical performance was assessed in different sample matrices. Known concentrations of the target analyte were spiked into deionized water and artificial serum to mimic simple aqueous and biological conditions, respectively. The spiked samples were analyzed in triplicate using the same protocol applied for standard solutions, and the sensor exhibited reproducible electrochemical responses in both matrices.

As presented in Table 2, recovery values ranging from 93% to 94% were obtained for the water samples, indicating minimal matrix interference and high detection accuracy. In artificial serum, recovery values between 80% and 85% were achieved, reflecting the greater complexity of the biological matrix while still demonstrating satisfactory analytical performance. Although moderate matrix interference was observed in serum samples, the obtained recoveries remained within acceptable ranges for bioanalytical applications. Potential approaches to mitigate matrix effects include dilution of biological samples, implementation of a standard-addition calibration strategy, and the use of blocking agents to minimize nonspecific adsorption. Such approaches may improve recovery and analytical accuracy in future bioanalytical applications. These findings confirm the potential applicability of the developed MIP/Fe_3_O_4_ NPs/BDD sensor for analyte detection in complex sample matrices.

**Table 2:** Determination of Malondialdehyde in Spiked Water and Artificial Serum

Spiked concentration (μM)	Recovery in water (%)	Recovery in serum (%)
0.05	93 ± 2	80 ± 5
0.1	94 ± 3	85 ± 5

## Conclusion

The present study successfully demonstrated the development of a sensitive and selective electrochemical MIP-based sensor for malondialdehyde detection using a dual-biopolymer matrix composed of chitosan and κ-carrageenan integrated with Fe_3_O_4_ nanoparticles on a boron-doped diamond electrode. The proposed sensing platform combined the recognition capability of molecular imprinting with the electrochemical advantages of Fe_3_O_4_ nanoparticles and the high conductivity and stability of the BDD substrate.

Optimization studies identified 75% chitosan, 25% κ-carrageenan, and 10 mg Fe_3_O_4_ nanoparticles as the optimal fabrication conditions for sensor performance. Under these conditions, the MIP/Fe_3_O_4_/BDD sensor exhibited excellent electrochemical response toward MDA, achieving a high sensitivity of 687.35 μA/μM, a low detection limit of 0.005 μM, and a linear detection range between 0.026 and 0.26 μM. The imprinting factor of 3.18 further confirmed the effectiveness of molecular imprinting in enhancing analyte recognition compared with the corresponding nonimprinted polymer.

The developed sensor also demonstrated good analytical performance, including excellent reproducibility (RSD = 4.6%), acceptable repeatability following multiple regeneration cycles (RSD = 7.5%), and high selectivity in the presence of structurally related compounds, biomolecules, and inorganic ions. In addition, the sensor maintained satisfactory recovery values in spiked water and artificial serum samples, supporting its applicability in complex matrices.

The originality of this work lies in the synergistic integration of a dual-biopolymer imprinted matrix with Fe_3_O_4_ nanoparticles on a BDD electrode, resulting in enhanced imprinting efficiency, improved electrochemical performance, and effective MDA recognition. Overall, the proposed platform represents a cost-effective, sustainable, and efficient sensing strategy for oxidative stress biomarker detection. Future investigations should focus on validation using real biological samples, evaluation of long-term storage and operational stability, and further development toward multiplexed and point-of-care diagnostic applications.

## Abbreviations

BDD: boron doped diamond
CV: cyclic voltammetry
DPV: differential pulse voltammetry
EIS: electrochemical impedance spectroscopy
Fe_3_O_4_: iron oxide
MDA: malondialdehyde
MIP: molecularly imprinted polymer
NIP: nonimprinted polymer
NP: nanoparticles
